# Recent advances in human cytomegalovirus: a comprehensive review of pathogenic mechanisms, virus-host interactions, and antiviral strategies

**DOI:** 10.3389/fimmu.2025.1636978

**Published:** 2026-01-16

**Authors:** Ying Xia, Ji Zhang, Xiaochuan Shui, Chao Wang, Shuaijie Zhang, Wenqi Chai, Yang Yang, Liang Shen, Chunhua Wang

**Affiliations:** 1 Department of Central Laboratory, Xiangyang Central Hospital, Affiliated Hospital of Hubei University of Arts and Science, Xiangyang, Hubei, China; 2 Department of Clinical Laboratory, Xiangyang Central Hospital, Affiliated Hospital of Hubei University of Arts and Science, Xiangyan, Hubei, China; 3 Department of Clinical Laboratory, Pathology and Blood Transfusion, The 991st Hospital of the Chinese People’s Liberation Army Joint Logistic Support Force, Xiangyang, Hubei, China; 4 Shenzhen Key Laboratory of Pathogen and Immunity, National Clinical Research Center for infectious disease, State Key Discipline of Infectious Disease, Shenzhen Third People’s Hospital, Second Hospital Affiliated to Southern University of Science and Technology, Shenzhen, China; 5 Department of Clinical Laboratory, Xiangyang No. 1 People’s Hospital, Hubei University of Medicine, Xiangyang, Hubei, China

**Keywords:** HCMV, pathogenesis, therapeutic drug, vaccine, virus-host interaction

## Abstract

Human cytomegalovirus (HCMV), recognized as the largest known DNA virus in the *Herpesviridae* family, is widely disseminated throughout the human population and poses a substantial threat to public health. HCMV is highly infectious. A majority of HCMV-infected individuals usually exhibit asymptomatic latent infections, while the immunocompromised or immunosuppressed populations infected with HCMV have high mortality. HCMV has long stood as a hot topic of research in the international virology community, and elucidating its pathogenic mechanisms is broadly regarded as a prerequisite for the development of vaccines and effective therapeutic drugs. This review presents a comprehensive and detailed analysis of the advances in the biological characteristics, pathogenesis, virus-host interactions, and the preventive and therapeutic strategies targeting HCMV infection. It will provide valuable insights and references for uncovering the pathogenic mechanisms of HCMV, identifying new drug targets, and developing potential immunotherapies.

## 1 Introduction

Human cytomegalovirus (HCMV) is a double-stranded DNA virus classified within the β subgenus of the *Herpesviridae* family. It is extensively distributed among the human population, with the majority of individuals being generally susceptible (1). Like other herpesviruses, once infected with HCMV, the individuals usually become lifelong carriers, and the virus can disseminate to multiple organs of the body via the bloodstream during the infection (2). The seropositivity rate of global HCMV infection is high, increasing with age and being inversely correlated with the economic level of a country or region (3–5). A majority of individuals with normal immune function usually exhibit asymptomatic latent infection or subclinical illness, such as fever, sore throat, rash, leukopenia, thrombocytopenia, and monocytosis. However, HCMV infection in immunocompromised or immunosuppressed individuals (organ transplant recipients, HIV-infected patients, and those undergoing cancer chemotherapy) can lead to severe diseases, such as hepatitis, neurological sequelae, retinitis, and pneumonia (6, 7). Primary HCMV infection during pregnancy may also cause chronic diseases in the fetus and newborn, such as hearing loss, brain malformations, and permanent disabilities, with severe cases potentially leading to death (13, 15). Moreover, HCMV infection is strongly linked to numerous other diseases, including atherosclerosis and cancer.

HCMV has diverse transmission routes, including horizontal transmission through blood, semen, saliva, urine, vaginal secretions, organ or bone marrow transplantation, and vertical transmission via the placenta to the fetus (3, 8, 9). It enters into host cells through membrane fusion or endocytosis, and enables to attack various types of cell, such as epithelial cells in the oropharynx, endothelial cells, hepatocytes, fibroblasts, smooth muscle cells, monocytes, macrophages, and dendritic cells (10). Of them, the infections of epithelial cells, endothelial cells, fibroblasts, and macrophages commonly trigger the lytic replication of HCMV, as the viral gene transcription is active to generate new viral genomes in these cells, and further form new infectious particles that spreading within the host or expanding to the population (11). However, HCMV infects undifferentiated myeloid cells, such as CD34^+^ hematopoietic progenitor cells (HPCs), the virus maintains a latent state (12). The process of HCMV infection is complex, involving the interactions between HCMV and host, so understanding the mechanisms of HCMV-host interactions is crucial for uncovering its pathogenesis.

Some host factors interacting with HCMV and their mediated signaling pathways have been uncovered to play crucial roles in the process of viral infection. For instance, the HCMV-encoded UL36 protein directly targets IRF3 to inhibit its interaction with STING/TBK1, thereby counteracting immune activation caused by anti-apoptotic mechanisms, and maintaining immune evasion (13, 14). During HCMV infection, the HCMV-encoded UL26 protein can prevent the phosphorylation of signal transducer and activator of transcription (STAT), antagonizing transcriptional activation induced by interferon α (IFN-α) or tumor necrosis factor α (TNF-α), and inhibiting the expression of antiviral genes (15–17). HCMV is capable of regulating gene expression of monocytes, suppressing their antigen-presenting and phagocytic functions, simultaneously enhancing the production of pro-inflammatory cytokines, which is further to weaken the abilities of monocytes to defend against pathogens and activate adaptive immune responses, leading to tissue damage and chronic inflammation (18). Furthermore, HCMV can also regulate the migratory properties of monocytes, promoting their transendothelial migration, thereby facilitating the systemic dissemination of the virus. Additionally, HCMV can influence the generation of reactive oxygen species (ROS), leading to damage in organs associated with HCMV infection, such as oxidative damage in the liver and ROS-mediated inflammatory responses in the lungs (19).

Although there are several antiviral drugs currently used to control HCMV infection, such as cidofovir and ganciclovir, the clinical treatment outcomes are less than ideal due to the drug resistance and toxic side effects (6, 20). Vaccination is a key strategy for preventing and controlling viral infections. Nonetheless, the development of an effective HCMV vaccine encounters significant challenges, including the extensive viral genome, difficulties in identifying immunogenic targets, antigenic variability among different strains, and the capacity of the host to evade immune responses. Therefore, a comprehensive understanding of HCMV biological characteristics and pathogenic mechanism is fundamental for the development of specific drugs and effective vaccines. This review discusses the advances in biological characteristics, pathogenesis, virus-host interactions, and antiviral strategies targeting HCMV infection, with the aim of providing new insights into HCMV pathogenesis, identifying new drug targets, and guiding the future development of potential immunotherapies for HCMV infection.

## 2 Biological characteristics of HCMV

**Figure 1:**
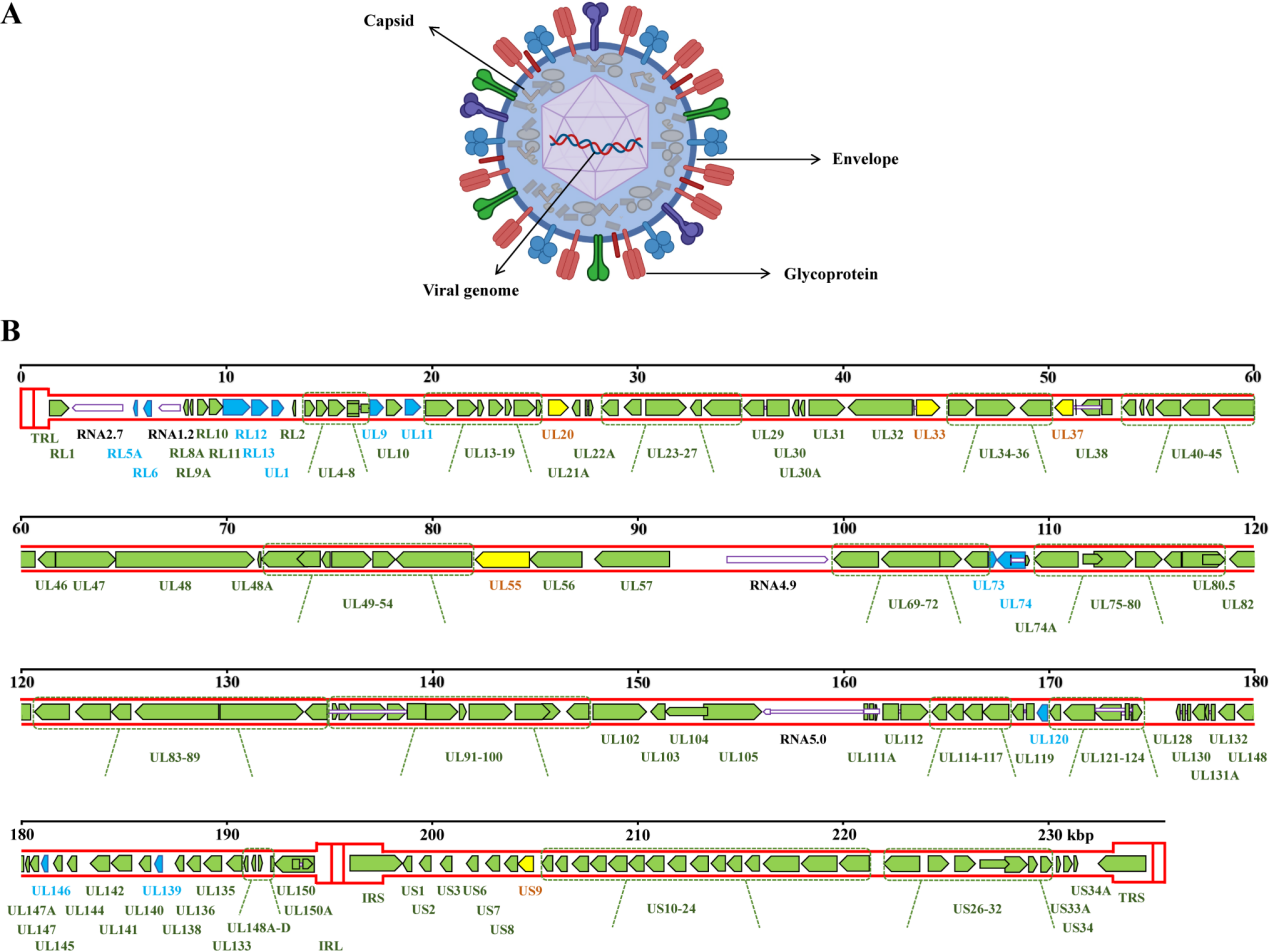
Schematic diagram of HCMV structure. **(A)** Schematic diagram of the HCMV particle. **(B)** Schematic diagram of the HCMV strain Merlin genome, which consists of the UL and US regions. The UL region has inverted repeat sequences TRL and IRL at both ends, while the US region has inverted repeat sequences IRS and TRS at both ends. Protein-coding regions are indicated by arrows, while non-coding RNAs are represented by thinner white arrows. Gene names are listed below. Introns are shown as thin white bars. The 12 genes (RL5A, RL6, RL12, RL13, UL1, UL9, UL11, UL73, UL74, UL120, UL146, and UL139) used for motif read-matching are shown in blue. RL13 and UL146 were also used for genotype read-matching. Additionally, 5 other genes (UL20, UL33, UL37, UL55, and US9) are used for genotype sequence analysis through alignment and are shown in yellow. Created with BioRender.com.

**Figure 2:**
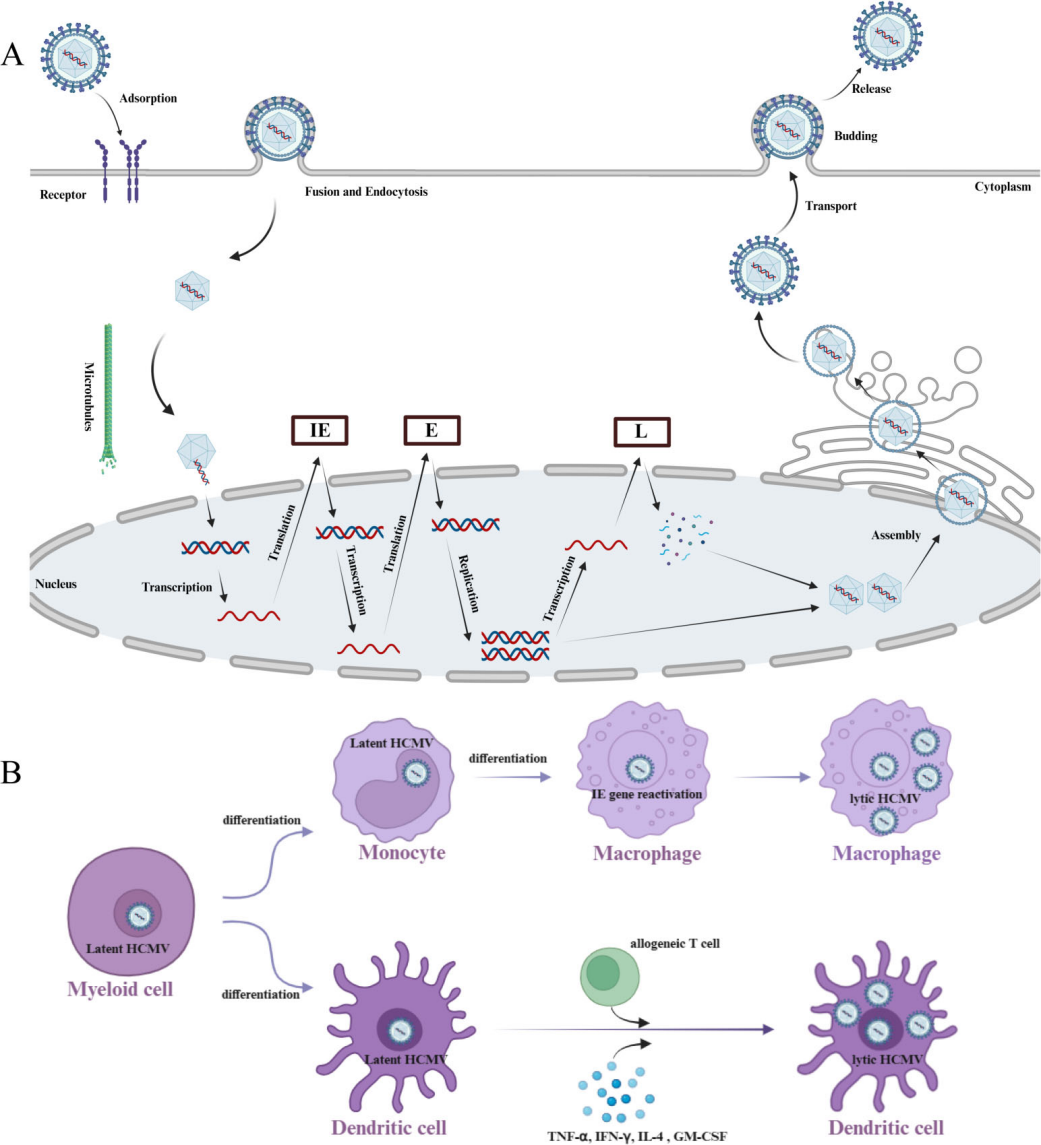
HCMV life cycle. **(A)** HCMV glycoproteins interact with specific cellular receptors to mediate endocytosis and membrane fusion. The nucleocapsid enters the nucleus via microtubules, and the linear DNA is released into the nucleus. Viral immediate early (IE) genes are transcribed and translated, followed by the transcription and translation of early (E) genes. The viral genome replicates in the nucleus, and late (L) proteins, primarily structural proteins, include the capsid, envelope, or membrane proteins, which are used for viral particle assembly and release. Subsequently, membrane proteins and glycoproteins assemble into mature viral particles in the endoplasmic reticulum and Golgi apparatus, and the virus is released into the extracellular space via budding. **(B)** Myeloid cells carrying latent HCMV can differentiate into monocytes and dendritic cells. Then the differentiation of monocytes into macrophages can induce the expression of HCMV IE genes, promoting the reactivation of latent HCMV; Dendritic cells, when stimulated by allogeneic T cells or inflammatory cytokines, can also lead to the reactivation of latent HCMV. Created with BioRender.com.

The viral particle of HCMV consists of viral genome and icosahedral capsid (**Figure 1A**). The HCMV genome is double-stranded linear DNA, measuring approximately 230–240 kb in length, which is the largest known genome among human herpesviruses (4). It consists of unique long (UL) and short (US) regions, with inverted repeat sequences located at both ends. This viral genome encodes more than 164 non-overlapping open reading frames (ORFs) and over 200 proteins (**Figure 1B**), which play crucial roles in viral replication and infection (21–24). The replication of HCMV depends on a temporal cascade gene expression mechanism, which is divided into three stages as the expressions of immediate early (IE) genes, early (E) genes, and late (L) genes (25). Once the HCMV enters into host cell through endocytosis or membrane fusion, the virus undergoes uncoating and releasing the viral genome. Then IE genes are transcribed, initially driven by activation of the major immediate early (MIE) locus, which consists of the core promoter, as well as proximal and distal enhancers (26). The products of IE genes transcription are IE1 (IE72) and IE2 (IE86), encoding by UL123 and UL122 genes, respectively. Studies have shown that IE72 and IE86 proteins play a critical role in the early stages of infection, especially IE86, which is essential for HCMV replication (27). IE86 drives the expression of viral genes as a transcriptional transactivator, and binds to its own promoter to suppresses the MIE promoter together with other auxiliary factors, forming a feedback loop to regulate promoter activity. After the expression of IE proteins, the synthesis of E gene products is initiated, including DNA polymerase (UL54), phosphotransferase (UL97), and terminases (UL51, UL52, pUL56, UL77, UL89, UL93, and UL104), which in turn promotes the replication and packaging of viral DNA. After the replication of the viral genome is completed, the expression of L genes is then activated. The proteins produced by these genes facilitate the formation of structural proteins essential for the assembly and release of viral particles, such as UL77, UL93, and pp150 (UL32) for capsid assembly, UL115 for envelope formation, and pp28 for the release of viral particles. Subsequently, the HCMV genome is packaged into the capsid, with the nucleocapsid initially forming a primary envelope at the inner nuclear membrane. It then crosses the nuclear membrane, uncoils at the outer nuclear membrane, and ultimately reaches the cytoplasm (4), where it curled within the endoplasmic reticulum-Golgi intermediate compartment, and finally the viral particles are released via budding (**Figure 2A**).

The HCMV infection is mainly divided into the lytic and latent phases, which largely depend on the type of infected cells. HCMV infects epithelial cells, endothelial cells, fibroblasts, or macrophages, the viral replication enters into the lytic phase. There are extensively viral gene expression and new viral genomes and infectious viral particles generated. The HCMV lytic infection is common observed in immunocompromised or immunosuppressed individuals, such as organ transplant recipients, HIV-infected patients, and those undergoing cancer chemotherapy. Especially, Over 90% of patients infected with HIV are seropositive for HCMV (28). After the highly active antiretroviral therapy (HAART) treatment for the HIV-HCMV-coinfected patients, the incidence of HCMV disease has decreased dramatically due to the reconstitution of humoral immunity against HCMV (29). Evidence confirms the accelerated aging and immunosenescence observed in HIV-HCMV-coinfected individuals during HAART is associated with HCMV replication (30, 31). However, HCMV infects undifferentiated myeloid cells, such as CD34^+^ HPCs, the virus enters into the latent phase (32). The viral gene (like MIE) transcription is suppressed and the viral replication is halted, thereby the virus remains dormant and no infectious viral particles are produced. Importantly, HCMV can switch from naturally latent to reactive state in infected myeloid cells, depending on the cell differentiation (**Figure 2B**). Differentiated myeloid cells may create conditions conducive to the reactivation of latent HCMV. What has now become apparent is that, once these myeloid cells differentiate to macrophages and dendritic cells (DCs), a fundamental change in their ability to support viral gene expression occurs. The macrophages differentiated from monocytes have been confirmed to result in reactivation of viral immediate early (IE) gene expression (33, 34). Furthermore, the full reactivation of infectious HCMV has been observed after CD34^+^ HPCs differentiating to mature DCs, which are stimulated by allogeneic T-cell or some cytokines (TNF-α, IFN-γ, IL-4 and GM-CSF) (35). Another study also reveals that lipopolysaccharides, TNF-α, and interferon-1β can trigger the activation of latent HCMV, which may suggest that inflammatory disease status can lead to the reactivation of HCMV from latency (36). Meanwhile, long-term HCMV infection stimulates a pro-inflammatory environment, which in turn supports the induction and exacerbation of chronic inflammatory diseases such as autoimmune diseases, some cancers, and cardiovascular diseases (37).

## 3 Pathogenic mechanisms of HCMV infection

### 3.1 Immune evasion

HCMV maintains its lifelong persistent infection through immune evasion pathways mediated by multiple immune regulatory factors, such as affecting the host immune responses, exerting anti-apoptotic effects, and facilitating viral replication. The HCMV IE1 affects the binding of STAT1 or STAT2 to the promoter of interferon-responsive genes and IE2 interferes with the binding of NF-κB to the IFN-β promoter to inhibit the transcription of immune effector (27); the proteins encoded by HCMV US1 and US11 genes are capable of mediating the endoplasmic reticulum-associated degradation (ERAD) pathway, to degrade the major histocompatibility complex class I (MHC-I) heavy chain, inhibiting antigen presentation and escaping immune surveillance (38); the US2 glycoprotein can inhibit the transport of MHC-II molecules, allowing the virus to evade CD4^+^ T cell immune responses (8); the HCMV PP65 protein can bind to the activating receptor NKp30 on NK cells, inhibiting NK cell activation (39). HCMV also can activate the oncogenic signaling pathways within the infected cells and directly transforms the cells, exhibiting anti-apoptosis function to evade the host immune responses. The HCMV UL36, as a caspase-activated inhibitor, can suppress Fas-mediated apoptotic pathway (31); the HCMV UL37 further enhances anti-apoptotic effects by preventing the recruitment of pro-apoptotic factors Bax and Bak to the mitochondria. Moreover, the HCMV UL38-encoded cell death inhibitory protein can prevent premature cell death, thereby enhancing viral replication; the HCMV US3 limits the transport of MHC I peptide complexes from the endoplasmic reticulum (ER), and the US6 protein blocks peptide transport into the ER, thus enhancing viral replication to evade the recognition of the immune system. Additionally, the HCMV UL23 enhances viral resistance to T cell-mediated cytotoxicity during infection, contributing to escape IFN-γ responses for virus.

### 3.2 Regulation of intracellular Ca^2+^ homeostasis, migration, and adhesion

The regulation of Ca^2+^ homeostasis can affect various cellular functions, including energy metabolism, cell proliferation and motility, transcriptional regulation, programmed cell death, and cellular responses to environmental changes and stress. Members of the transmembrane Bax-inhibitor 1 motif-containing (TMBIM) protein family are transmembrane proteins that regulate various adaptive cellular responses by modulating intracellular Ca^2+^ homeostasis. The family of HCMV US12 genes consists of 10 consecutive genes (US12-US21), with US21 encoding a seven-pass transmembrane endoplasmic reticulum (ER)-resident viral pore protein. US21 shares high homology with two transmembrane Bax inhibitor motif (TMBIM) family members, Bax-inhibitor 1 (BI-1) and Golgi anti-apoptotic protein (GAAP). US21 is a viral regulatory factor that modulates intracellular Ca^2+^ homeostasis, stimulates cell migration, and enhances adhesion. It decreases ER Ca^2+^ storage content, thereby protecting cells from apoptosis (40). Furthermore, US21 activates Calpain 2 and interacts with Talin-1, a key protein component of the focal adhesion complex, to mediate cell migration and adhesion. The deletion of the US21 gene weakens the migratory ability of HCMV-infected cells, and the Ca^2+^ channel activity of US21, as well as its interaction with Talin-1, are crucial for regulating cell migration and adhesion. Silencing Talin-1 or inhibiting Calpain 2 can block this process (41).

### 3.3 Carcinogenesis

Similar to Epstein-Barr virus (EBV), Kaposi’s sarcoma-associated herpesvirus, human papillomavirus (KSHV), hepatitis B virus (HBV), hepatitis C virus (HCV), human T-cell leukemia virus type 1 (HTLV-1) and Merkle cell polyomavirus (MCPyV), an oncogenic role of HCMV has been highlighted in which the virus directly transforms primary cells, it therefore might be defined as the eighth human oncovirus (42). Recent studies have reported that HCMV infection is associated with the occurrence of certain human malignancies, such as glioblastoma, neuroblastoma, colon cancer, breast cancer, ovarian cancer and prostate adenocarcinoma (43–47). The oncoviruses promote tumorigenesis by various mechanisms, potentially directly affecting host cell DNA, disrupting cell cycle regulation, inhibiting tumor suppressor genes, or activating oncogenes (48). HCMV supports tumor growth through the encoding of viral proteins and other means. The HCMV genome contains several oncogene-associated genes, such as IE1, IE2, US28, and UL76. Additionally, numerous other important genes increase the ability of HCMV to infect tumor cells, enhancing the invasiveness of malignant tumors. HCMV infection can activate multiple critical signaling pathways associated with cancer, such as the EZH2-Myc, JAK-STAT3, and PI3K/AKT/mTOR pathways. HCMV encodes several homologs of cytokines, including UL146 (IL-8-like) and UL111a (vIL10), with HCMV vIL10 being secreted in tumor cells and associated with the presence of TAMs (tumor-associated macrophages) in related cancers (49).

### 3.4 Molecular pathways of HCMV infection

The host immune response to HCMV infection plays a crucial role in the pathogenic mechanisms of the virus. Following viral invasion, the host mucosal immune system serves as the first line of defense, preventing HCMV from attaching to and entering susceptible cells. The host innate immune system, including phagocytes, IFN, and pro-inflammatory cytokines, employs various mechanisms to counter the viral infection. Meanwhile, the host adaptive immune response is mediated by B lymphocytes and T lymphocytes in coordination with other immune mechanisms, specifically neutralizing and eliminating the virus. The ability of HCMV to reactivate from its latent phase is closely related to changes in cellular signaling pathways, which subsequently triggers the differentiation of HPCs.

#### 3.4.1 EZH2-Myc signaling pathway

**Figure 3:**
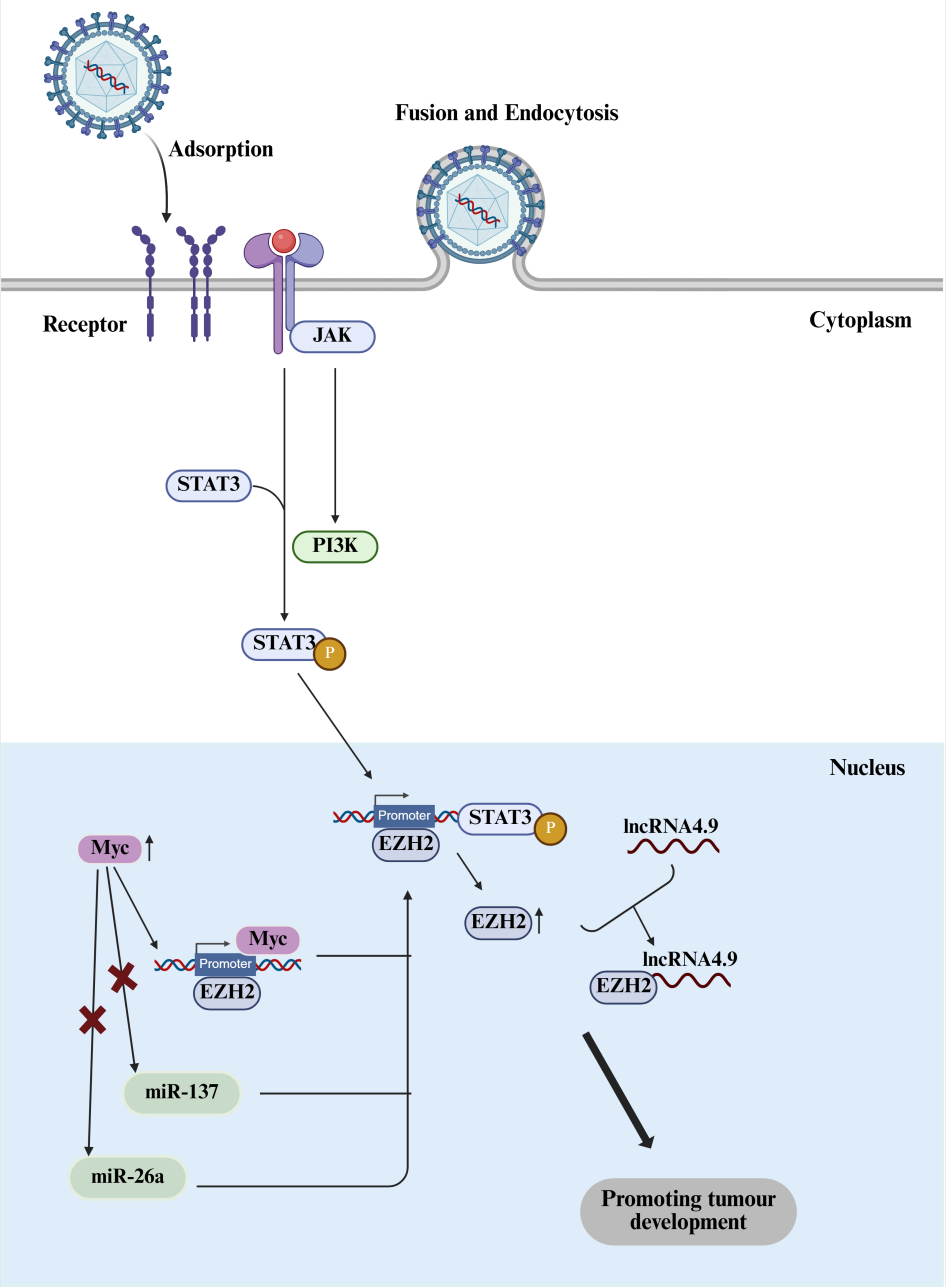
EZH2-Myc signaling axis. Myc acts as an upstream regulator of EZH2, promoting the transcription of EZH2 by binding to its promoter or inhibiting its negative regulators (such as miR-26a and miR-137), thereby jointly driving cellular transformation and the maintenance of stem cell characteristics. Created with BioRender.com.

The mechanisms by which viruses induce cancer are not limited to direct involvement in cellular transformation, they can also create a favorable microenvironment for cancer development by triggering chronic inflammation. EZH2 is an important epigenetic regulator, highly expressed in various cancers, which is considered as a potential target for cancer therapy. The Myc gene is abnormal in over 50% of human cancers and plays a role in regulating various cellular processes. Different oncogenic viruses, such as HPV, HTLV, and KSHV, activate the EZH2-Myc signaling axis through their specific mechanisms, thereby affecting tumor initiation and progression. HCMV infection is closely associated with the occurrence and development of breast cancer and glioblastoma. HCMV can regulate tumor processes and support the formation of polyploid giant cancer cells (PGCCs). In human mammary epithelial cells (HMEC), which are infected with HCMV isolated from clinical breast cancer samples, the expression levels of Myc and EZH2 are elevated, leading to the formation of polyploidy. In high-risk HCMV-infected HMECs, HCMV-transformed HMEC cells (CTH cells) exhibit morphological heterogeneity, undergo the giant cell cycle, and maintain long-term replication of HCMV (50). EZH2 plays a key role in the proliferation, transformation, stem cell characteristic maintenance, and the HCMV replication of CTH cells. In CTH cells, both EZH2 and Myc expression are upregulated, and they display characteristics of epithelial-mesenchymal transition (EMT) and stem cell traits (50). In breast cancer tissues, the presence of PGCCs correlates with elevated expression of EZH2 and Myc, and in the case of HCMV infection, there is a strong positive correlation between the expression of EZH2 and Myc, as well as the number of PGCCs. HCMV strains isolated from glioblastoma tissues, when infecting human astrocytes (HAs), induce cell transformation and form cells with glioblastoma-like characteristics (CEGBCs) (**Figure 3**). These cells exhibit multiple malignant features, including a dedifferentiated state in two-dimensional culture with mesenchymal transformation (PMT) characteristics, and the ability to form spheroidal structures and invade surrounding tissues in three-dimensional culture (51). In HCMV-detected positive glioblastoma tissues, the expression of EZH2 and Myc is significantly upregulated, and the expression levels of both are positively correlated. The presence of HCMV is closely associated with the expression levels of both. The activation of EZH2 and Myc plays an important role in tumor initiation, stem cell maintenance, and tumor progression. After both acute and chronic HCMV infections, EZH2 acts as a downstream target of HCMV-induced Myc upregulation (52).

#### 3.4.2 PI3K/Akt/mTORC1 signaling pathway

HCMV spreads to the host through peripheral blood monocytes and can activate a non-canonical Akt signaling pathway upon infection, which subsequently activates mTORC1 and promotes the translation of anti-apoptotic proteins, aiding for the replication of virus. The PI3K/Akt signaling pathway is a crucial negative regulator of HCMV replication. During latency, the PI3K/Akt pathway can limit the viral replication; during lytic infection, the virus inhibits Akt phosphorylation and activity through UL38. The application of PI3K or Akt inhibitors enhances viral replication, indicating that Akt signaling exerts varying influences on viral replication differently at various stages of HCMV infection. HCMV infection also induces significant changes in mRNA translation, inhibiting mTOR (a component of mTORC1) significantly reduces protein synthesis. The SUnSET labeling experiment indicates that monocytes infected with HCMV or UV-inactivated HCMV continue to synthesize a large amount of proteins up to 72 hours. HCMV infection activates mTORC1, leading to phosphorylation of 4E-BP1 and eEF2K, which promotes the initiation and elongation of translation. While the myeloid growth factors can activate mTOR phosphorylation, they do not effectively activate 4E-BP1 and eEF2K. Inhibition of mTORC1 with rapamycin suppresses HCMV-induced protein synthesis, but has little effect on growth factor-induced mRNA translation, suggesting that mTORC1 plays a critical role in regulating translation in monocytes. Furthermore, HCMV infection also promotes SIRT1 expression. Inhibition of SIRT1 leads to cell death in infected cells but has little impact on uninfected cells, indicating that SIRT1 is crucial for the survival of infected cells. SIRT1 deacetylates Akt, promoting its activation and sustaining the HCMV-induced Akt/mTORC1 signaling pathway. Inhibiting SIRT1 reduces phosphorylation levels of Akt, mTOR, and other proteins, decreasing the expression of anti-apoptotic factors and inhibiting protein synthesis. HCMV infection stimulates the PI3K/Akt/mTORC1 signaling pathway to increase SIRT1 expression, which further enhances Akt and mTORC1 activity, forming a positive feedback loop (53).

**Figure 4:**
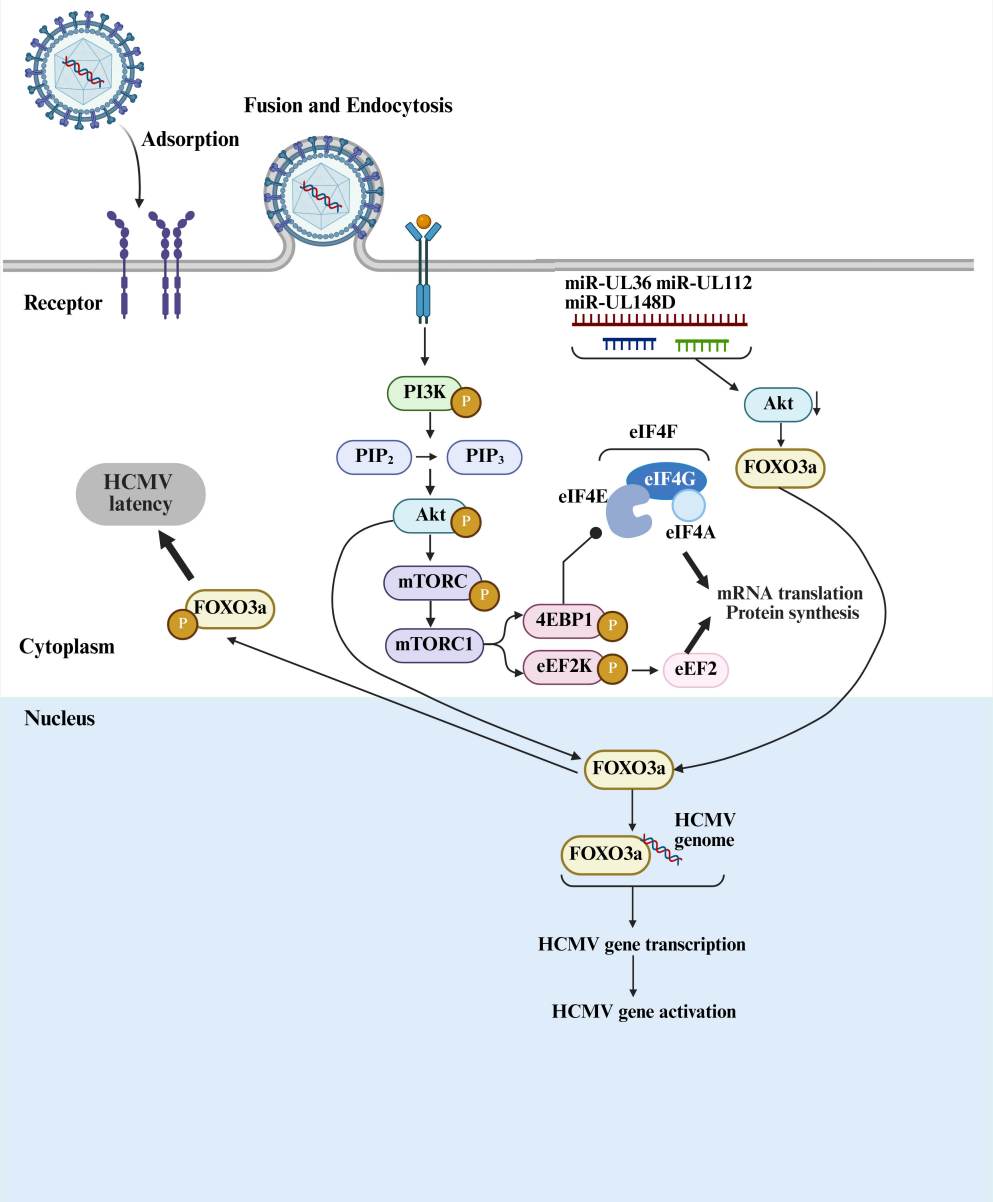
PI3K/Akt/mTORC1 signaling pathway. After HCMV infection, the PI3K/Akt signaling pathway is activated, leading to the phosphorylation of Akt at Ser473. Once activated, Akt phosphorylates FOXO3a, causing its translocation from the nucleus to the cytoplasm, which inhibits its transcriptional activity and maintains the latent state of HCMV. HCMV-encoded miR-UL36, miR-UL112, and miR-UL148D downregulate Akt through a non-canonical mechanism, relieving Akt’s inhibitory effect on viral reactivation. In this case, dephosphorylated FOXO3a binds to specific regions of the viral genome in the nucleus, driving the transcription of IE genes and promoting HCMV reactivation. Following HCMV infection, Akt phosphorylates mTOR and activates mTORC1. Once activated, mTORC1 further phosphorylates 4E-BP1, facilitating the assembly of the eIF4F complex, which sustains translation activation and ensures viral protein synthesis. Created with BioRender.com.

Additionally, HCMV microRNAs, including miR-UL36, miR-UL112, and miR-UL148D, can downregulate Akt expression, affecting downstream signaling pathways and promoting the activation and nuclear translocation of FOXO3a, thereby inducing the expression of relevant viral transcripts, which is crucial for HCMV reactivation from latency (13). Moreover, inhibiting Akt signaling enhances HCMV reactivation, as viruses lacking the aforementioned miRNAs fail to reactivate effectively *in vitro* and *in vivo*, whereas inhibiting Akt can partially restore reactivation (**Figure 4**).

#### 3.4.3 Interferon type I signaling pathway

**Figure 5:**
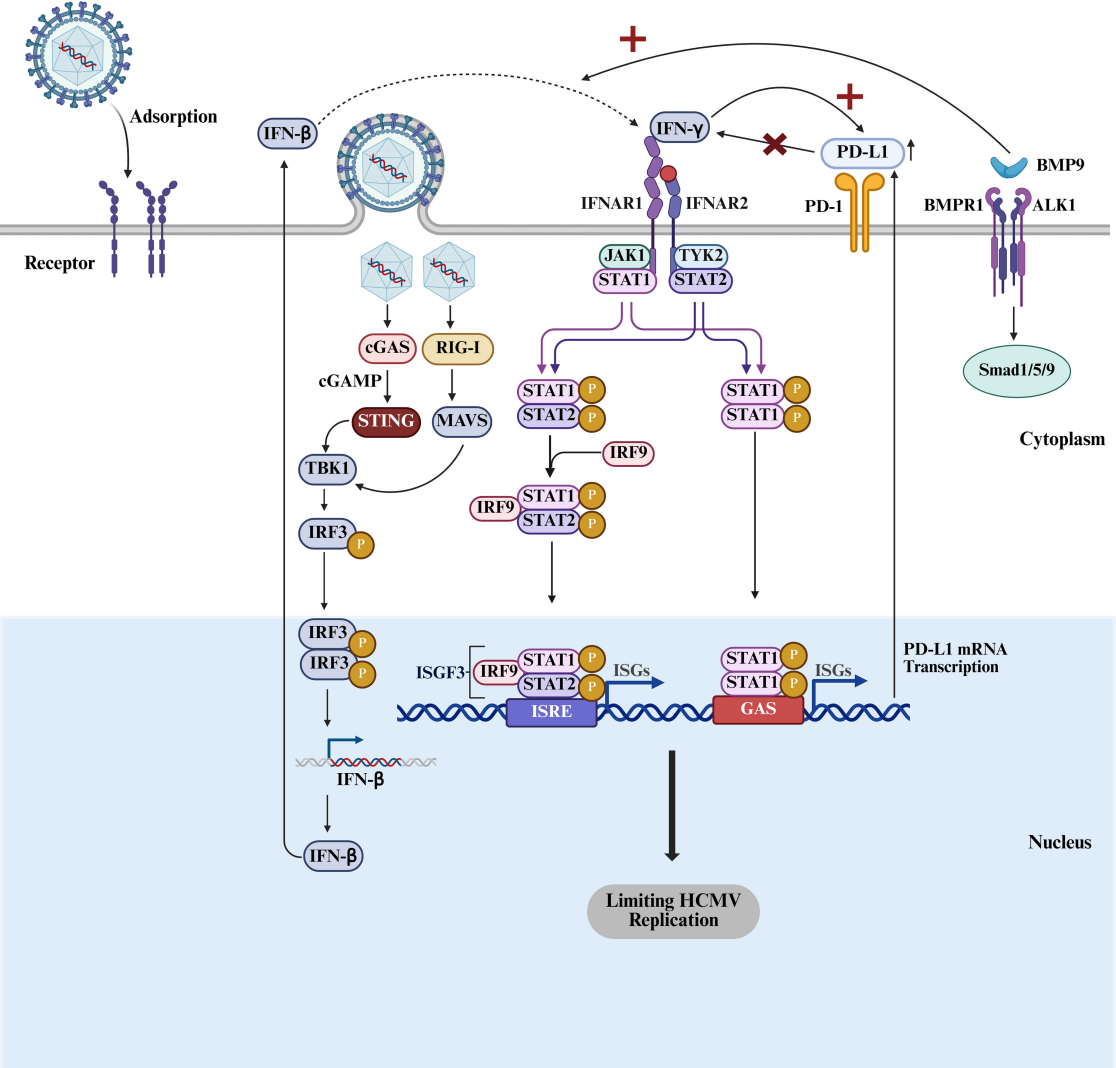
IFN signaling pathway. After HCMV infection, the host recognizes viral nucleic acids through pattern recognition receptors RIG-I and cGAS, activating mitochondrial antiviral signaling protein MAVS and the STING pathway, thereby inducing the expression of IFN-β. Upon binding to its receptor, IFN-β activates the JAK-STAT pathway, which further induces the expression of interferon-stimulated genes (ISGs). These ISGs limit HCMV replication through mechanisms such as inhibiting viral protein synthesis and promoting viral degradation. BMP9 enhances the antiviral immune response of the host by activating the SMAD1/5/9 signaling pathway and cooperating with IFN-β to promote the expression of ISGs. IFN-γ promotes the expression of ISGs by activating the JAK-STAT pathway, thereby inhibiting HCMV replication. Moreover, the sustained release of IFN-γ after HCMV infection drives the high expression of PD-L1. The viral envelope protein UL23 directly activates the PD-L1 gene promoter, promoting PD-L1 expression. PD-L1, by binding to the PD-1 receptor on T cells, inhibits the secretion of IFN-γ by T cells, thereby evading the host immune response. Created with BioRender.com.

The interferon type I (IFN-I) signaling pathway is a significant component of the host’s innate immune response to antiviral defense, capable of inducing the expression of IFN-stimulated genes (ISGs). In this process, mitochondria play a crucial regulatory role. MFN1 is a critical regulator of mitochondrial fusion. After infection with HCMV in THP-1 cells and human monocytes, the expression of IFN-I related molecules and MFN1 increased, mitochondrial fusion was enhanced, and fission was reduced. Silencing MFN1 inhibits mitochondrial fusion and reduces the production of IFN-I induced by HCMV. There is an interaction between MFN1 and MAVS (54); silencing MFN1 does not affect MAVS expression but inhibits the redistribution of MAVS induced by HCMV infection, thereby reducing IFN-I production. It suggests that MFN1 is essential for MAVS redistribution and IFN-I production. Leflunomide, the MFN1-specific agonist, can induce MFN1/2-dependent mitochondrial fusion and increase the expression of MFN1, p-TBK1, and the mRNA levels of related genes, thereby enhancing HCMV-induced IFN-I production (**Figure 5**). These indicate that HCMV-induced IFN-I production is mediated by MFN1 (14).

#### 3.4.4 IFN-γ signaling pathway

T cell immune responses play a crucial role in the defense against HCMV infections, while HCMV employs various strategies to resist the host immune response. For instance, the UL23 protein of HCMV helps the virus evade the IFN-γ response (55). IFN-γ is primarily secreted by T cells, and the replication levels of HCMV UL23 knockout mutants are significantly reduced after co-culturing with T cells, while wild-type virus is better able to resist T cell-mediated cytotoxicity. Overexpression of UL23 enhances HCMV replication in co-culture with T cells, indicating that UL23 boosts the virus resistance to T cell-mediated killing. Blocking the IFN-γ signaling pathway significantly alleviates the decrease in viral titers caused by UL23 deletion, while blocking other T cell cytotoxic pathways does not yield similar effects. The molecular mechanism of that is the UL23 can inhibit the T cell activation and proliferation, promote the apoptosis of T cells, thereby suppressing IFN-γ secretion. Comparative RNA sequencing of human fibroblasts infected with UL23-deficient HCMV and wild-type HCMV reveals the significant differences in PD-L1 gene expression. After knocking out PD-L1, the inhibitory effect of UL23 on T cell IFN-γ secretion is markedly reduced. Addition of purified recombinant PD-L1 protein to the co-culture system with UL23 deletion can reduce the inhibition of IFN-γ secretion. Meanwhile, The addition of PD-L1 antibodies can counteract the suppression of T cell function by UL23. UL23 can activate the PD-L1 gene promoter, and upregulate PD-L1 expression. During HCMV infection, PD-L1 expression is positively correlated with UL23 (**Figure 5**). Blocking PD-L1 function or expression can eliminate the enhancing effect of UL23 on wild-type HCMV viral titers, indicating that HCMV relies on UL23 to evade T cell-mediated cytotoxicity by regulating the PD-L1 signaling pathway (56).

#### 3.4.5 BMP9-mediated IFN pathway

HCMV infection of human fibroblast cells (HFF-1) induces the secretion of BMP9. BMP9 synergizes with IFN-β to significantly enhance antiviral effects and reduce the number of HCMV IE1-positive cells. The co-stimulatory effect of BMP9 and IFN-β strengthens the IFN-β-mediated signaling pathway, leading to enhanced transcription levels of certain ISGs while simultaneously decreased the expression of related negative regulatory factors. The enhancement of BMP9-induced ISG expression depends on the activation of the IFNAR signaling pathway. BMP9 enhances the phosphorylation of STAT1 stimulated with IFN-I (IFN-β or IFN-α), promoting the transcription of related genes, but has no effect on the signaling pathway mediated by IFN type II (IFN-γ) (57). The HCMV US18 and US20 proteins, components of the viral particles, inhibit BMP9-mediated signaling but do not affect IFN-I signaling (**Figure 5**). Overexpression of these two proteins in HFF-1 cells suppresses BMP9 signaling activation, while HCMV mutants lacking these proteins show increased sensitivity to the antiviral effects of IFN-β (58).

#### 3.4.6 RAF1-MAPK signaling pathway

**Figure 6:**
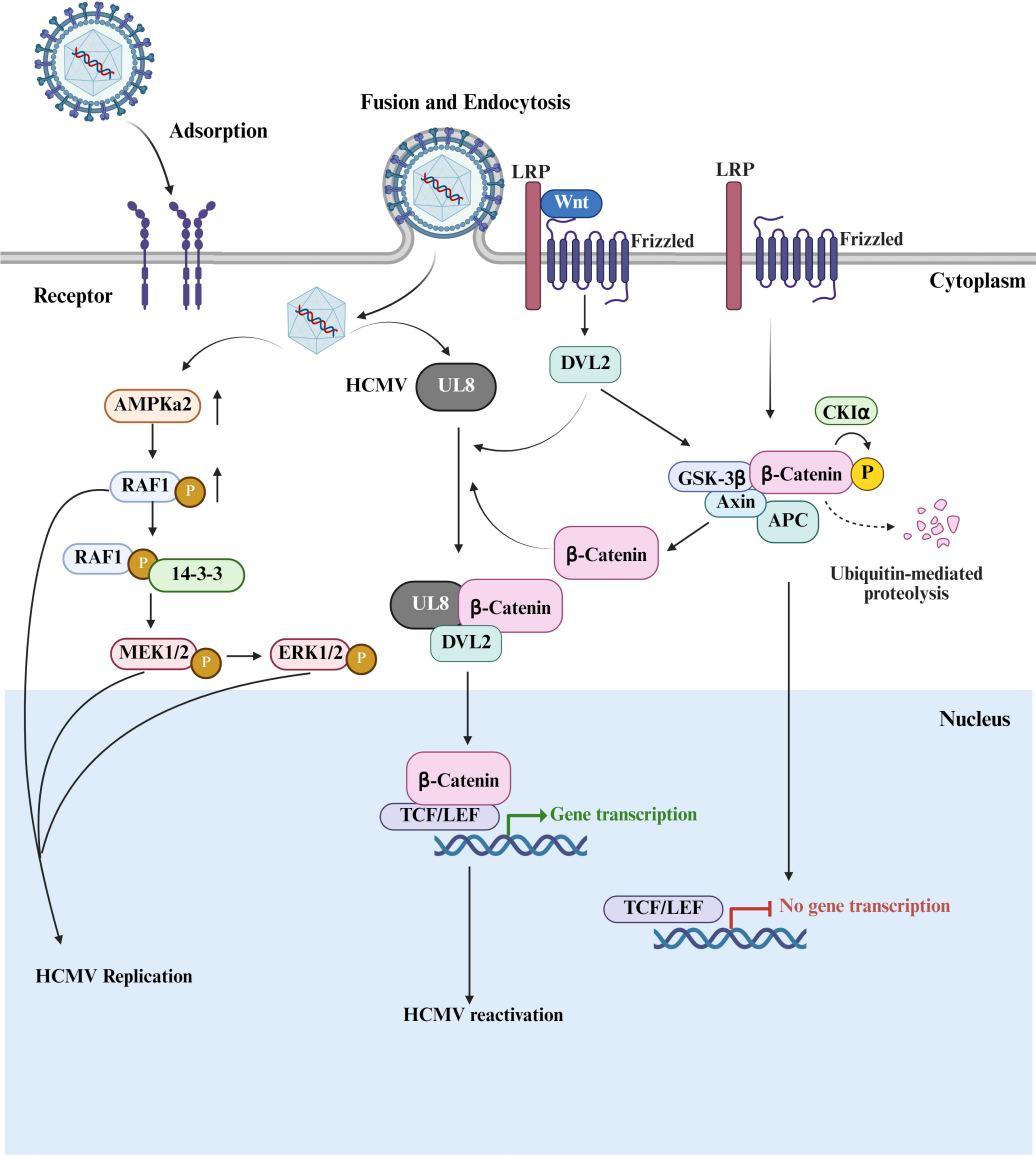
RAF1-MAPK and Wnt/β-catenin signaling pathways. The left panel shows that HCMV infection induces the activation of AMPKα2, which leads to the phosphorylation of RAF1, enhancing the binding of RAF1 to the 14-3–3 scaffold protein. This results in an increase in the expression of RAF1 downstream targets P-ERK and P-MEK, thereby promoting viral replication. The right panel illustrates that when the Wnt signaling pathway is in a dormant state, β-catenin binds to the APC/Axin/GSK-3β complex and, after being phosphorylated by CKIα, is degraded through the ubiquitin-proteasome pathway. The Wnt signal binds to the Frizzled/LRP receptors, recruits DVL2 protein, and inhibits the degradation of β-catenin, which in turn upregulates the expression of UL8. UL8, in collaboration with DVL2, activates β-catenin and facilitates its entry into the nucleus, where it binds to TCF/LEF transcription factors, thereby activating downstream target genes and reactivating HCMV replication. Created with BioRender.com.

RAF1 is a key component in the mitogen-activated protein kinase (MAPK) cascade, and the AMPK acts as a regulatory factor for RAF1. HCMV infection induces AMPK activity (59). HCMV infection leads to specific changes in the phosphorylation of RAF1 protein. In the early stages of infection, the phosphorylation level at the S338 site of RAF1 increases and then decreases, a process unaffected by AMPK. However, the phosphorylation level at the S621 site of RAF1 significantly increases during infection, and this increase can be blocked by AMPK inhibitors. HCMV-induced RAF1-S621 phosphorylation enhances the binding of RAF1 to the 14-3–3 scaffold protein, which is a key step in activating RAF1. Overexpressing RAF1 or constructing the non-phosphorylated mutant RAF1-S621A show that phosphorylation at the S621 site is crucial for RAF1 stability and its interaction with 14-3-3. Treatment with RAF1-specific inhibitors, such as Regorafenib or Sorafenib, reduces the accumulation of viral DNA and proteins, as well as the production of infectious viral particles is suppressed, with lower phosphorylation levels of RAF1 downstream targets P-MEK and P-ERK. These results indicate that inhibiting RAF1 significantly impacts the HCMV infection process (**Figure 6**). Overall, RAF1 plays an important role in HCMV infection, and its activity is regulated by AMPK (60).

#### 3.4.7 Wnt/β-catenin signaling pathway

HCMV can establish latent infection in CD34^+^ HPCs. The Wnt/β-catenin signaling pathway plays a key role in the normal function and development of hematopoietic cells (61). The CD34^+^ HPCs infected with HCMV-ΔUL8 (UL8 deleted) results in significantly fewer plaques compared to that infected with wild-type virus, although viral DNA levels remain unchanged. The viral DNA copies increase in spleen and liver tissues of mice infected with wild-type HCMV, while that does not observed in HCMV-ΔUL8-infected mice, indicating the UL8 is crucial for HCMV reactivation but not for the establishment of latent infection. BioID-MS combined with co-immunoprecipitation (Co-IP) experiments reveals that the UL8 interacts with β-catenin in the Wnt pathway. The application of Wnt signaling antagonist DKK-1 inhibits virus reactivation. Both UL8 and β-catenin possess PDZ-binding domains, which are essential for their interaction. BioID-MS analysis shows that the UL8 is located at the PDZ domain-containing DVL2 proximal region, and UL8 can only bind to DVL2 when its PDZ-binding domain is functional. Mutating these binding domains or blocking the interaction by the Pen-N3 peptide disrupts the formation of the β-catenin-UL8-DVL2 complex. HCMV infection reduces total β-catenin levels, and cells infected with HCMV-ΔUL8 exhibit even lower levels of active β-catenin (**Figure 6**). Overexpression of UL8 increases active β-catenin levels and promotes transcriptional activation of Wnt target genes, such as c-myc and survivin (Birc5), while mutations in the PDZ-binding domain weaken this effect. Importantly, disrupting the formation of the UL8-β-catenin-DVL2 complex inhibits viral reactivation (62).

## 4 HCMV-host interactions

HCMV infection relies on the virus-host interaction, and its lytic replication efficiency is regulated by the interactions between viral and host proteins (**Table 1**).

**Table 1:** Host proteins interacting with HCMV.

Viral gene transcriptional stages	Viral gene/ protein	Host interactor	Biological functions of host proteins	References
IE	IE	YBX1	Regulation of mRNA translation	(63)
IE	IE1、IE2	PLSCR1	Inhibition of tumorigenesis, promotion of cell apoptosis, and assistance in myeloid cell differentiation	(64)
IE	UL26	PIAS1	Regulation of immune signaling pathways, involvement in the viral infection process, influence on cellular response to cytokines, regulation of cholesterol metabolism-related gene expression	(65)
IE	UL26	ISG15	Regulation of immune signaling, antiviral activity	(66)
IE	US28	RhoGEF	Regulation of Rho GTPases, influencing cell migration, differentiation, and other cellular processes	(67)
IE	US28	CX3CL1	Regulation of immune cell migration and adhesion, modulation of immune signaling	(68)
IE	UL36	IRF3	Activation of immune response, regulation of cell apoptosis	(69)
IE	UL138	UAF1-USP1 complex	DNA damage repair, influence on the viral infection process	(70)
E	UL97	RFX7	Influence on neural progenitor cell proliferation and migration	(71)
E	UL97	Cyclin H, CDK7	Regulation of CDK7 activity, influence on gene transcription, involvement in transcriptional regulation related to viral infection	(72)
E	UL97	Cyclin types T1/B1/H	Cell cycle regulation	(73)
E	UL97	14-3-3	Involvement in signal transduction, protein localization, and stability regulation in various cellular processes	(74)
E/L	UL4-UL6	ZAP	Antiviral activity, regulation of intracellular mRNA stability	(75)
L	UL44	L1 ORF2p	Catalysis of retrotransposition, involvement in DNA damage response, influence on viral replication, activation of innate immune signaling via the cGAS/STING pathway	(76)
L	UL44	UBC9	SUMOylation of proteins	(77, 78)
L	gpUL4	TRAIL	Induction of cell apoptosis, involvement in NK cell activation, participation in antiviral immunity	(79)
L	UL82	DDX5	AGR2 transcriptional repressor	(80)
L	UL82	Rae1	mRNA nuclear export regulation	(81)

### 4.1 Host proteins interacting with HCMV IE1/2

The RNA-binding protein YBX1 promotes its binding to polyribosomes and translation efficiency by binding to HCMV IE mRNA. Knockout of YBX1 significantly reduces viral protein expression and viral particle production, but restoring YBX1 expression can reverse this effect, indicating that YBX1 plays a critical regulatory role in the effective translation of HCMV IE RNA (63). Moreover, PLSCR1, a key regulatory factor of the IFN-I pathway, is also a restriction factor of HCMV. PLSCR1 interacts with HCMV IE1 and IE2 to reduce the formation of CREB-IE2 and CBP-IE2 complexes and inhibit the CREB-mediated transcriptional activation. PLSCR1 negatively regulates HCMV replication by suppressing the transcription of the viral MIE and early promoters. Deletion of PLSCR1 enhances viral plaque formation, viral titers, and MIE protein expression, whereas its overexpression can inhibit these processes, suggesting the PLSCR1 is a key host factor that plays a defensive role in the IFN-I antiviral pathway by blocking HCMV transcription (64).

### 4.2 Host proteins interacting with HCMV UL26

HCMV UL26 regulates the host immune response via multiple mechanisms. Its C-terminal domain inhibits the phosphorylation of STAT1, STAT2, and STAT3, and forms a complex with immune regulatory proteins such as PIAS1. Mutations in the C-terminal of UL26 enhance STAT phosphorylation. PIAS1 knockout suppresses viral replication and upregulates the expression of ISGs, but has a minimal effect on UL26-deleted strains, indicating that PIAS1 primarily promotes HCMV infection by limiting ISGs expression (65). Early HCMV infection triggers the expression of ISG15 and an increase in ISGylation levels. The UL26 interacts with ISG15 in a dual manner: on one hand, it is covalently modified by ISG15 at the K136/K169 sites through ISGylation, which weakens its inhibition of the NF-κB pathway and promotes viral replication; on the other hand, it binds to ISG15, UBE1L, and Herc5 to suppress ISGylation enzyme activity. HCMV counters the host defense via a dual mechanism, as inhibiting ISG15 transcription by IE1 and blocking ISGylation by UL26. Meanwhile, ISG15 limits HCMV infection by modifying viral/host proteins, establishing an immune evasion system centered around UL26 (66).

### 4.3 Host proteins interacting with HCMV US28

HCMV encodes multiple putative G-protein coupled receptors (GPCRs). Of them, the US28 acts as a viral chemokine receptor, expressed during both HCMV latent and lytic phases. It promotes cell migration, transformation, and plays a key role in viral latency and reactivation. Using the US28-TurboID system in conjunction with mass spectrometry, several proteins interacting with US28 are identified, including PDZ-RhoGEF, p115-RhoGEF, ROCK1, Src, and ERK. It is found that the US28 signaling complex significantly overlaps with the RhoA signaling pathway, and RhoGEFs are highly enriched in the US28 proximity markers. Small molecule inhibitors, such as Rhosin and Y16, used to block the interaction between RhoA and GEFs, inhibits the RhoA activation and significantly weakens the US28 signal (67). US28 binds to CX3CL1 to form a CX3CL1-US28 complex, and the complex is associated with Gi proteins to form two non-productive conformations: the classical state (C-state) and the orthogonal classical state (OC-state). Through the formation of a ‘Gi sink’ that sequesters Gi proteins and suppresses their GTP hydrolysis activity, blocking host chemokine signaling, thereby aiding HCMV escape from host immune surveillance (68).

### 4.4 Host proteins interacting with HCMV UL36

In the innate immune response, the cGAS-STING pathway can recognize viral DNA and activate IRF3, inducing IFN-I to exert antiviral effects. Apoptosis restricts viral infection by eliminating infected cells. HCMV UL36, a caspase-8 inhibitor, can block the extrinsic apoptotic pathway, and caspase-8 also inhibits IRF3 activation. UL36 blocks the phosphorylation and activation of IRF3 by inhibiting the binding of IRF3 to TBK1 and STING (69). Cell experiments confirm the necessity of wild-type UL36 for viral replication, revealing its dual function of maintaining infection by synergistically inhibiting apoptosis and innate immune signaling.

### 4.5 Host proteins interacting with HCMV UL138

The UL132-UL150 genes of HCMV have little effect on viral replication in fibroblasts but have a greater impact on the replication level of HCMV in hematopoietic stem cells and endothelial cells. The expression of UL138 restricts the replication of HCMV, while knockout of UL138 leads to the restoration of HCMV replication in HPCs. So the UL138 is of great significance for maintaining viral latency. The immunoprecipitation-mass spectrometry (IP-MS) assay has found that UL138 interacts with UAF1 and USP1. UAF1 is a scaffold protein, which plays a key role in the activity of ubiquitin-specific peptidases (USPs), including USP1. UL138 plays a key role in maintaining the level of pSTAT1 during infection. pSTAT1 is located in the nucleus of HCMV-infected cells, which is the site of viral DNA replication and transcription. It binds to the viral genome and affects the expression of UL138. Inhibition of USP1 prevents the establishment of latency, leading to increased viral genome replication and enhanced production of viral precursor proteins (70).

### 4.6 Host proteins interacting with HCMV UL97

UL97 is a CDK-like kinase encoded by HCMV, exhibiting structural and functional similarities to host cell cycle-dependent kinases (CDKs). During HCMV infection of primary neural progenitor cells (NPCs), UL97 activates the SOCS3 promoter by phosphorylating the transcription factor RFX7, leading to a significant upregulation of SOCS3 expression. Knockdown of either UL97 or RFX7 blocks the HCMV-induced enhancement of SOCS3 transcription, and phosphatase treatment confirms that UL97 regulates SOCS3 expression through RFX7 phosphorylation. Furthermore, HCMV infection increases the expression of SOCS3 in the fetal and postnatal mouse brain. Overexpression of SOCS3 in mice hinders NPCs migration, thus affecting normal fetal brain development, revealing a key pathway by which the virus hijacks host transcriptional regulatory networks to interfere with neurodevelopment (71). UL97 can phosphorylate CDK substrates, thereby substituting for CDK functions. During HCMV infection, CDK7 interacts with UL97 and Cyclin H to form a ternary complex that jointly regulates RNA polymerase II (RNAP II) activity and trans-stimulates CDK7, enhancing its kinase activity to modulate transcription in infected cells (72). UL97 specifically interacts with Cyclin T1, H, and B1. Cyclin T1 binds to the N-terminus of UL97, promoting UL97 homooligomerization, which may be involved in the assembly of the viral nucleocapsid or the formation of genomic replication complexes. In HCMV-infected human fibroblasts, the Cyclin H expression decreases; knockdown of Cyclin H results in reduced UL97 kinase activity, inhibiting viral replication. Cyclin H enhances UL97 kinase activity by binding to its N-terminus, thereby promoting viral genome replication and late gene expression. Cyclin B1 binds to the C-terminal kinase domain of UL97 and phosphorylates the host retinoblastoma protein (Rb) by UL97 kinase activity, inducing a G1/S phase cell cycle block, providing a favorable microenvironment for viral replication. The functions of Cyclin T1 and B1 can be compensated, while Cyclin H plays a core regulatory role in viral replication (73). The Ser-13 phosphorylation site on UL97 interacts with 14-3–3 protein β, ϵ, η, γ, and θ subtypes, and this interaction depends on UL97 kinase activity, enhancing its stability. The Ser-13 mutation (S13A) reduces UL97 protein stability without affecting its kinase activity or viral replicated capability in fibroblasts (74).

### 4.7 Host proteins interacting with HCMV UL4-UL6

Cellular antiviral factors can inhibit viral replication by recognizing viral nucleic acids, one of which is the zinc-finger antiviral protein (ZAP). ZAP restricts the replication of the HCMV TB40/E strain by recognizing CpG dinucleotide-rich regions in viral RNA. ZAP can interact with the RNA of HCMV UL4-UL6. In HCMV-infected cells, the expression of ZAP decreases over time. ZAP-S (Short isoform of ZAP) in infected cells partially depends on the activation of the IFN-I signaling pathway. Knockdown of ZAP results in an increase in HCMV titer, with elevated expression of viral proteins such as IE2, UL57, and pp65. Knockdown of ZAP-L (Long isoform of ZAP) or ZAP-S expression shows that the inhibition of ZAP-S expression significantly increases viral titer, suggesting that ZAP-S plays a critical role in restricting HCMV replication (75).

### 4.8 Host proteins interacting with HCMV UL44

Viruses can hijack host components to promote replication during the replication cycle of virus. HCMV infection upregulates the expression of transcription factors YY1 and RUNX3, and enhances the chromatin accessibility of the L1 promoter region. The increased expression of L1 transposons facilitates HCMV replication. The HCMV DNA polymerase subunit UL44 is a core protein of the L1 ribonucleoprotein (RNP) complex. UL44 can directly interact with the host LINE1 ORF2p, inducing a DNA damage response in the HCMV replication compartment. This alleviates replication stress and accelerates viral DNA replication (76). UL44 also interacts with the host E3 SUMO ligase PIAS3, which acts as an E3 ligase to promote SUMOylation at the conserved K410 site of UL44. This modification directs UL44 to co-localize with PML proteins in the host nuclear domain 10 (ND10) structures, thus restricting UL44 to the ND10 subnuclear region and inhibiting HCMV DNA replication (77). In contrast, the K410R mutation of UL44 disrupts SUMOylation and ND10 localization, significantly enhancing viral replication. Additionally, it has been reported that UL44 interacts with the SUMO conjugating enzyme UBC9. The K410 mutation (K410A) significantly reduces SUMOylation levels but does not affect protein stability or nuclear localization. However, the K410 mutation significantly enhances viral DNA synthesis and progeny virus production. SUMOylation inhibits HCMV replication by targeting UL44 (78).

### 4.9 Host proteins interacting with HCMV gpUL4

The HCMV UL4 gene encodes a highly glycosylated secretory protein, gpUL4, which is primarily localized in the endoplasmic reticulum and does not serve as a component of viral particles. As a late-stage expressed protein, gpUL4 dose-dependently inhibits NK cell activation, specifically by suppressing NK cell degranulation and inducing the production of IFN-γ and TNF-α. This inhibitory effect is donor-independent. NK cells play a crucial role in controlling HCMV infection, and HCMV has evolved various mechanisms to evade NK cell recognition. The interaction between gpUL4 and TRAIL proteins has been confirmed. TRAIL exists in both soluble and membrane-bound forms. gpUL4 can prevent TRAIL from binding to its receptors, acting as a soluble TRAIL decoy receptor, thereby inhibiting TRAIL-induced apoptosis and NK cell activation (79).

### 4.10 Host proteins interacting with HCMV UL82

Recent studies have found a closely correlation between HCMV infection and poor prognosis in colorectal cancer (CRC), with multiple viral proteins such as UL82, UL42, and UL117 detectable in tumor tissues. Among them, UL82 shows the highest positive rate, and its expression level is significantly correlated with patient survival rates. The HCMV UL82 is a phosphorylated protein with a nuclear localization signal (NLS), which is rapidly transported to the nucleus after viral infection of host cells. Anterior gradient protein 2 (AGR2), a protein disulfide isomerase located in the endoplasmic reticulum (ER), primarily involves in the folding of nascent proteins. It exhibits upregulated expression in various cancers. HCMV infection or overexpression of UL82 can enhance AGR2 expression, while knockdown of AGR2 reverses the proliferative and cell cycle-promoting effects of UL82. DEAD-box helicase 5 (DDX5), a transcriptional repressor of AGR2, interacts with UL82, reducing the inhibitory effect on AGR2 (80). Furthermore, the UL82-AGR2 axis affects the levels of nucleotide metabolism-related enzymes, promoting nucleotide metabolism and thereby driving DNA replication and cell proliferation. Additionally, UL82 has been confirmed to interact with Rae1, suggesting that this interaction regulates host mRNA metabolism, affecting viral replication and immune evasion (81). This provides partial evidence for the mechanism by which HCMV hijacks host factors to promote its own replication.

## 5 Anti-HCMV drugs

Research into therapeutic drugs for HCMV infection has made notable advancements, and various agents have been identified that can inhibit HCMV infection, such as viral DNA polymerase inhibitors, nucleoside and nucleotide analogs, pyrophosphate analogs, and terminal enzyme inhibitors (**Table 2**). Currently, the U.S. FDA has approved six drugs for the treatment of HCMV infections: Ganciclovir and its prodrug Valganciclovir, Letermovir, Maribavir, Cidofovir, and Foscarnet (6). Among them, oral Valganciclovir is first converted to Ganciclovir in the body, which is further converted to its active triphosphate form to exert its antiviral effects by inhibiting viral DNA replication. Foscarnet and Cidofovir exert their antiviral effects by inhibiting the DNA polymerase activity of HCMV, while Maribavir produces antiviral effects by inhibiting the protein kinase of HCMV. Letermovir targets the inhibition of HCMV terminase complex. Although these drugs show inhibitory effects against HCMV, they still face numerous issues such as adverse reactions and drug resistance (82, 83).

**Table 2:** Summary of drugs and vaccines.

Types	Names	Characteristics	Side effects	References
Drugs and Inhibitors	Ganciclovir	2’-Deoxyguanosine synthetic nucleoside analog, inhibits HCMV viral DNA polymerase	Infertility correlation, carcinogenic and teratogenic effects, bone marrow toxicity, neutropenia, nephrotoxicity	(20, 84–92)
	Valganciclovir	Prodrug of Ganciclovir, rapidly converted to Ganciclovir after oral administration		
	Letermovir	HCMV terminal enzyme inhibitor	Gastrointestinal symptoms such as nausea, abdominal pain, fatigue, cough, headache, and peripheral edema	(93, 94, 128)
	Maribavir	Benzimidazole L-ribonucleoside, inhibits HCMV protein kinase UL97	Taste disturbances, nausea, diarrhea, vomiting, and fatigue	(20, 94, 102)
	Cidofovir	Monophosphate nucleoside analog, Converted to diphosphate form via intracellular phosphorylation, competitively inhibits the incorporation of deoxycytidine triphosphate (dCTP) into viral DNA, preventing viral DNA chain elongation	Neutropenia, renal toxicity, fever, chills, headache, rash, nausea, vomiting, fatigue, and alopecia	(96–98, 129)
	Foscarnet	Pyrophosphate analog, Non-competitively inhibits CMV DNA polymerase, prevents pyrophosphate group cleavage from deoxynucleoside triphosphates, interferes with DNA chain synthesis	Mild to moderate renal toxicity, hypophosphatemia, intermittent muscle spasms, decreased hemoglobin, and nausea	(95, 99–101)
	Pyrido[2,3-b]Pyrazine	Non-nucleoside inhibitors, targeting HCMV DNA polymerase, binding to different sites on the enzyme to inhibit its Function, blocking viral DNA synthesis and replication	–	(102)
	LDC4297	Targeting Host CDK7 Kinase, Disrupting Viral Replication Cycle	–	(103, 104)
	HN0141	DNA terminase complex inhibitor, blocking viral genome cleavage and packaging	Mild clinical indicator abnormalities, including elevated alanine aminotransferase (ALT) and triglycerides	(105)
	ARP101	Inhibits HCMV by inducing the non-classical Keap1-Nrf2 Pathway	–	(106)
	Valspodar	Inhibits host cell ATP-binding cassette (ABC) transporters	–	(107)
	N-arylpyrimidinamine	Targeting the immediate early phase of HCMV infection	–	(108)
Vaccines	Triplex	Encoding three CMV antigens, UL83 (pp65), UL123 (IE1-exon4), and UL122 (IE2-exon5), with recombinant modified vaccinia ankara (MVA) virus	Fatigue, myalgia, and headache	(112, 113)
	pp65-CRM197+gH-CRM197	pp65 is an important target for HCMV-specific CTLs, gH plays a crucial role in virus-cell surface receptor binding, cell fusion, and virus entry; CRM197 is a non-toxic mutant of diphtheria toxin, activating CD4+ T cells through heterogeneous Th1 and Th2 cytokines, leading to B cell activation and antibody regulation, constructed into a peptide-CRM197 Vaccine	–	(114)
	gH/gL/pUL128/pUL130/pUL131	Engineered AD169 Strain Modified to Express the CMV Pentamer Complex, Incorporating Chemically Controlled Protein Stability Switches	Injection site pain (muscle injection); injection site erythema (intradermal injection); common systemic adverse events including fatigue and headache	(115, 116)

### 5.1 Ganciclovir

Ganciclovir is a synthetic nucleoside analog of 2’-deoxyguanosine, with the antiviral mechanism is to inhibit viral DNA polymerase (84). During HCMV infection, the drug is converted to Ganciclovir 5’-monophosphate by the viral kinase encoded by HCMV UL97, and is further catalyzed by cellular kinases (such as deoxyguanosine kinase, guanylate kinase, and phosphoglycerate kinase) to form Ganciclovir diphosphate and triphosphate (20). Ganciclovir triphosphate competitively inhibits viral DNA polymerase and inserts into the replicating HCMV genome, causing premature termination of viral DNA synthesis, thereby preventing further viral replication and reducing viral loads. However, mutations in the viral kinases or DNA polymerase genes may lead to HCMV resistance to Ganciclovir. A study has reported that seven intestinal obstruction patients with an average age of 61, infected with HCMV infection, have treated with Ganciclovir experienced 2 deaths, which might be associated with the drug’s side effects or resistance leading to reduced efficacy (85). Though the oral Ganciclovir has been approved to prevent initial episodes of HCMV retinitis and other forms of HCMV disease among individuals with advanced AIDS by FDA (86), the drug adverse effects are a major determinant impacting the therapeutic choice for individual patients in clinical practice (87). Ganciclovir is typically used for the treatment of HCMV infection and prophylaxis in solid organ transplant recipients, though dose-dependent cytopenias, particularly leukopenia, are frequently observed. Accordingly, it is used more judiciously in hematopoietic stem cell transplant (HCT) recipients owing to concern for graft toxicity (82).

### 5.2 Valganciclovir

Valganciclovir is a prodrug of Ganciclovir, which is rapidly converted to Ganciclovir after oral administration (88). It has high oral bioavailability, and is typically used to treat a wide range of manifestations of the infection, including the treatment of HCMV infection in immunocompromised individuals, such as the treatment for HCMV retinitis in HIV-infected patients (89). Meanwhile, Valganciclovir is also used as the primary/secondary prophylaxis in transplant recipients (prior to the availability of letermovir), especially preventing the HCMV disease in high-risk transplant recipients (90, 91). In a study, 51 kidney transplant recipients were enrolled, with 31 being HCMV seropositive and 20 seronegative. Valganciclovir was initiated after kidney transplantation when the patient’s creatinine clearance rate exceeded 20 mL/min, with the dosage adjusted according to the creatinine clearance rate. The median duration of Valganciclovir use was 113 days (range: 37 to 329 days), with only one patient showing asymptomatic HCMV DNA viremia, which was attributed to the failure to adjust the Valganciclovir dosage according to the creatinine clearance rate. During the follow-up after discontinuation of treatment, 12 patients (23.5%) developed HCMV DNA viremia, with 2 patients testing positive for HCMV DNA multiple times without clinical symptoms. The median follow-up time after Valganciclovir prophylaxis was 252 days (range: 45 to 425 days), and no HCMV-related diseases were observed during this period. It indicates that Valganciclovir is highly effective in preventing HCMV disease in HCMV-seropositive kidney transplant recipients (92).

### 5.3 Letermovir

Letermovir is the first HCMV terminase complex inhibitor, preventing the packaging of the viral genome by inhibiting the viral terminase complex (93). This drug is primarily used for the prevention of HCMV infection following organ transplantation and allogeneic hematopoietic stem cell transplantation (HSCT). Letermovir can be administered orally or intravenously, has a high protein binding rate, and is metabolized in the liver via the UGT1A1/1A3 enzyme system, with excretion mainly through feces. Letermovir is a substrate for multiple drug transporters, including P-glycoprotein (P-gp), organic anion transporting polypeptide 1B1/3 (OATP1B1/3), and breast cancer resistance protein (BCRP) (94). Additionally, Letermovir is a moderate inhibitor of CYP2C8 and CYP3A, a moderate inducer of CYP2C19, a moderate inhibitor of renal OAT3 transporters, and a moderate inhibitor of hepatic OATP1B1/3. In pharmacokinetic studies of HSCT patients and healthy volunteers, Letermovir showed no significant differences, but the bioavailability in HSCT patients significantly decreased, possibly due to mucosal inflammation. UL56 is a key protein in the HCMV terminase complex, crucial for the binding of the terminase complex to Letermovir. Mutations in the amino acid sequence of UL56 protein 229–369 lead to HCMV resistance to Letermovir (82).

### 5.4 Maribavir

Maribavir is a drug that inhibits the UL97 kinase and is also a benzimidazole L-ribonucleoside. It works by competitively inhibiting the binding of ATP to pUL97, thereby preventing HCMV DNA replication, capsid formation, and nuclear egress (94, 95). This drug is administered orally, with common side effects including gastrointestinal symptoms. In a study involving 352 patients who had undergone solid organ transplantation or HSCT, those treated with Maribavir had a higher HCMV clearance rate compared to patients treated with other drugs, such as Ganciclovir, Foscarnet, and Cidofovir. In patients with primary HCMV infection following HSCT, two groups of 241 patients each received either Maribavir or Valganciclovir for 8 weeks, with a 12-week follow-up period. At 21 days of treatment, the rate of resistance mutations in the Maribavir group was 10%, significantly higher than the 2.5% in the Valganciclovir group, and resistance appeared earlier (median time 56 days vs 90 days). Resistance was associated with mutations in the UL97 gene, including T409M and H411Y, with one case of the G343A mutation, which showed resistance to both Maribavir and Valganciclovir. In the Maribavir treatment group, 23 patients switched to alternative antiviral drugs, and 74% of those with resistance achieved viral clearance, with only one treatment failure. However, in the preventive treatment setting, some patients carrying the UL97 T409M mutation showed reactivation of HCMV after receiving Maribavir therapy (20).

### 5.5 Cidofovir

Cidofovir is a nucleotide analogue of monophosphate, used as a first-line treatment for adenovirus infections and as a second-line treatment for resistant herpesvirus infections. It is converted into its diphosphate form through intracellular phosphorylation, which then competitively inhibits the incorporation of deoxycytidine triphosphate (dCTP) into viral DNA, thereby preventing the elongation of the viral DNA chain (96). Cidofovir has a broad-spectrum antiviral effect, inhibiting the replication of multiple viruses, but prolonged use can lead to nephrotoxicity. During treatment with Ganciclovir, due to resistance phenomena caused by mutations in the UL97 gene or mutations in the UL54 gene of HIV-infected HCMV strains, Cidofovir often proves to be more effective as an alternative drug (97). For patients with HIV-associated cytomegalovirus retinitis, Cidofovir’s advantage lies in its long intracellular half-life, which allows for intermittent intravenous administration (98).

### 5.6 Foscarnet

Foscarnet is a pyrophosphate analogue that non-competitively inhibits CMV DNA polymerase, preventing the cleavage of pyrophosphate groups from deoxynucleotide triphosphates, thus interfering with DNA chain synthesis (95). It exhibits inhibitory effects on a wide range of herpesviruses. Due to its low oral bioavailability (12-22%), it is typically administered intravenously (99). In patients with HIV-associated cytomegalovirus retinitis, 85% to 95% of patients experience lesion healing or stabilization after 2 to 3 weeks of treatment. For gastrointestinal diseases caused by HCMV, the complete or partial relief rate with Foscarnet ranges from 57% to 95%. Furthermore, Foscarnet significantly reduces circulating HIV antigen levels in HIV-infected patients with HCMV infection (100). The drug’s adverse effects primarily include nephrotoxicity, bone marrow suppression, mucosal ulcers, hypokalemia, hypomagnesemia, and hypocalcemia. In a study involving 10 HIV patients with HCMV retinitis, 9 patients showed improvement, while 1 patient could not be evaluated due to retinal detachment (101).

### 5.7 Other inhibitors

In addition to the six FDA-approved drugs mentioned earlier, recent studies have identified several novel small-molecule inhibitors against HCMV, such as Pyrido[2,3-b]Pyrazine (102), LDC4297 (103, 104), HN0141 (105), ARP101 (106), Valspodar (107), N-arylpyrimidinamine (108) and ruxotinib (109). However, these small-molecule inhibitors are still in the cell-based or animal testing stages, and considerable time and researches are required before they can be applied in clinical practice (110).

## 6 HCMV vaccines

Due to the general susceptibility of the population to HCMV, the development of an HCMV vaccine is of great significance for preventing and reducing HCMV infection. Currently, research primarily focuses on developing various types of HCMV vaccines using HCMV envelope glycoprotein gB, pentameric complex, and the envelope protein pp65 as immunogens. These vaccines mainly include live vaccines (such as attenuated live vaccines and recombinant virus vaccines) and inactivated vaccines (such as subunit vaccines, RNA vaccines, virus-like particle vaccines, and DNA vaccines) (111). However, although some vaccines have progressed clinical trials, none of the HCMV-related vaccines have been clinically approved to date due to the unclear immune protection mechanisms of HCMV and the absence of appropriate animal models.

In a Phase I clinical trial of a recombinant attenuated vaccinia virus (MVA) vaccine (Triplex) containing three dominant antigens, HCMV L83 (pp65), UL123 (IE1-exon 4), and UL122 (IE2-exon 5), healthy adults who received two doses of different vaccine amounts did not experience any vaccine-related severe adverse events, indicating that the vaccine has good safety and tolerance in the adult population. In addition, the Triplex vaccine was able to induce strong and long-lasting HCMV-specific T cell responses in the inoculated population, without triggering non-specific immune responses against Epstein-Barr virus (EBV) (112). HCMV reactivation may increase the morbidity and mortality of HCT recipients. In a Phase II clinical trial, 102 HCMV-seropositive HCT recipients were vaccinated with the Triplex vaccine. No vaccine-related severe adverse events occurred on days 28 and 56 post-vaccination, demonstrating the vaccine’s good safety profile. The incidence of HCMV reactivation in the vaccine group was 9.8%, significantly lower than the 19.6% in the placebo group, though the difference was not statistically significant. Furthermore, the level of pp65-specific T cells in the vaccine group was significantly higher than that in the placebo group, with a higher proportion of effector memory T cells, indicating that the recombinant vaccine has good tolerance and can effectively induce and enhance HCMV-specific immune responses (113).

In the study of a peptide vaccine constructed by screening HCMV pp65 and gH antigen epitopes and conjugating them with the CRM197 carrier (pp65-CRM197, gH-CRM197, and pp65-CRM197+gH-CRM197), it was found that all three vaccines were able to induce CD8^+^ T cells to secrete high levels of IFN-γ and TNF-α, and CD4^+^ T cells to secrete high levels of IFN-γ, IL-2, and IL-4, with these cytokine levels significantly higher than in the HCMV inactivated treatment group. In addition, all three vaccines induced higher levels of neutralizing antibodies, with the highest neutralizing antibody titers observed in the pp65-CRM197+gH-CRM197 vaccine (114). These vaccines promote both innate and adaptive immune responses through various synergistic mechanisms.

The replication-defective HCMV vaccine V160 is a gH/gL/UL128/UL130/UL131 pentameric vaccine. In a Phase I clinical trial, participants received three doses of V160, The results indicated that V160 was well tolerated and induced HCMV-specific neutralizing antibodies (NAbs) and T cell responses, similar to the immune responses induced by natural infection (115). However, the phase IIb clinical trial data showed that the vaccine’s immune protection rate was only 44.6%, and it did not significantly reduce the incidence of viral infection compared to the placebo group. Therefore, the study of this vaccine was terminated (116).

## 7 Discussion

HCMV is the largest known DNA virus in the human virome, with its genome exhibiting significant genetic diversity and complexity. Through long-term interaction and evolution with its host, HCMV has developed various strategies to evade the host immune system, promoting its own growth and replication (27, 41). By manipulating the host cell cycle, HCMV escapes immune system surveillance and even interferes with cytokine signaling pathways to maintain its persistent presence in the host (4). During infection of epithelial cells, the virus enters the lytic phase, with increased transcriptional activity of viral genes and the production of infectious viral particles. However, when infecting CD34^+^ HPCs (hematopoietic progenitor cells) or CD14^+^ monocytes, the virus enters the latent phase, where gene transcription is suppressed, and no viral particles are produced (53, 117). Furthermore, HCMV targets host-related proteins and signaling pathways through multiple viral proteins to initiate evasion mechanisms, forming complex interactions with the host immune system to maintain lifelong infection. For instance, IE1 escapes immune responses by inhibiting STAT1/2 binding to IFN response gene promoters; IE2 interferes with the binding of NF-κB to the IFN-β promoter (15, 70, 118, 119); US1 and US11 degrade MHC-I heavy chains via the ERAD pathway, and US2 inhibits the translocation of MHC-II molecules, hindering antigen presentation (120, 121); IE proteins suppress apoptosis through p53-dependent or independent pathways, while UL36-UL38 also inhibit apoptosis; UL23 enhances viral resistance to T-cell cytotoxicity and antagonizes the IFN-γ response, among other mechanisms (56). These interaction networks reveal the molecular mechanisms by which HCMV uses multiple strategies to exploit host resources, evade immune surveillance, and drive the infection process, thereby promoting its replication. Notably, HCMV is a potential oncogenic virus, with genes such as IE1, IE2, US28, and UL76 confirmed to be oncogenes that activate tumor-associated signaling pathways. Some HCMV genes, such as vIL10, can also enhance viral infectivity and tumor invasiveness in tumor cells. These mechanisms collectively contribute to tumorigenesis and progression (122). Future research should explore more interactions between viral and host factors, with a focus on the complex interplay between HCMV-encoded proteins and host factors, further elucidating the intricate regulatory networks involved in apoptosis, immune evasion, and efficient replication.

Existing antiviral therapies have many limitations, as the six drugs approved for clinical treatment target the same stage of DNA replication, which can lead to increased drug resistance and adverse effects (123). Utilizing technologies such as AI design and protein structure analysis to discover additional potential HCMV targets is key to addressing the current issue of antiviral drug resistance. Moreover, because the HCMV lifecycle relies on various host cell factors to complete its replication and infection processes, developing drugs that target critical host cell restriction factors has become an important research direction for the future. Currently, to enhance the efficacy and reduce the adverse effects of treatment for HCMV infection, several studies have explored the combination therapy regimens for the treatment of HCMV infection. Letermovir demonstrates additive effects when combined with Ganciclovir, Valganciclovir, Foscarnet, and Cidofovir, it also shows no interaction when used in conjunction with anti-HIV drugs, providing *in vitro* evidence for combination therapy in transplant recipients with multidrug-resistant HCMV infections and HCMV-HIV co-infected patients (124). Cidofovir combined with either Ganciclovir, Foscarnet or Acyclovir has been found additive to synergistic inhibition of HCMV replication. The suppression of CMV replication was obtained at lower drug concentrations when the drugs were combined than when the drugs were used alone. These may indicated that use of combinations in the therapy of HCMV infections can enhance drug efficacy, reduce toxicity and, possibly, to diminish the risk of emergence of drug-resistant virus strains (125). Maribavir shows additive effects when combined with Ganciclovirin against multidrug-resistant HCMV infections *in vitro (*126). The immunomodulatory agents (such as leflunomide and everolimus) and anti-HCMV immunoglobulin may act as supplementary treatment options when used alongside direct anti-HCMV drugs(such as Foscarnet and Ganciclovir) (127). Although combination therapy has shown initial antiviral effects against HCMV, further in-depth clinical studies are needed for validation.

It is known that vaccines are considered the best option for preventing HCMV infection, while there no HCMV vaccine has been approved to date. The main challenges in HCMV vaccine development include its complex biological characteristics, immune evasion mechanisms, and low protection rates in clinical trials (111). It remains unclear why current HCMV vaccines in clinical trials have failed to induce sufficient immune protection, which is a critical issue to address in future research. Additionally, the high variability among different HCMV strains necessitates that vaccine design consider both conserved components and strain-specific differences, as well as safety (7).

There are several challenges in developing therapies that precisely target both latent and active HCMV infections. Firstly, it is difficult to accurately differentiate the infection status. During latent HCMV infection, the virus remains at a low level within hematopoietic stem cells, endothelial cells, and other cell types, without releasing obvious viral replication markers. Existing detection technologies (such as viral load testing and antigen detection) struggle to differentiate between low-level latency and early active infection, which can lead to overtreatment or delayed treatment. Secondly, there is insufficient selectivity in treatment targets. Latent infection relies on host cell signaling pathways to maintain its latent state, while active infection focuses on key enzymes involved in viral replication. The targets for these two states differ significantly. Current drugs primarily target the viral replication processes during active infections and lack specificity for drugs aimed at activating or eliminating latent viruses. Thirdly, there is a dilemma in balancing drug resistance and toxicity. Long-term use of traditional anti-HCMV drugs (such as Ganciclovir) can induce viral resistance mutations, while treatment for latent infection requires prolonged intervention, increasing the risk of cumulative drug toxicity (such as bone marrow suppression and nephrotoxicity). Fourthly, host immune status interferes with treatment outcomes. Immunocompromised populations (such as transplant recipients and HIV patients) are at high risk for HCMV infection, and their immune deficiencies lead to frequent transitions in infection status. Additionally, the synergistic mechanisms between immunomodulators and antiviral drugs are not fully understood, complicating the individualized design of treatment strategies.

In summary, this detailed analysis reviews the latest research advances regarding pathogenic mechanisms, HCMV-host interactions and treatment methods. It aim is to explore the reasons for HCMV latent infection from a pathogenic perspective, integrating existing evidence to deepen the understanding of HCMV pathogenesis, and provide valuable evidence for HCMV prevention and clinical treatment. With continuous advancements in structural biology and AI-related biological technologies, it is believed that more HCMV-related antiviral targets will be identified in the future, providing new tools to combat HCMV infection.
